# Chronic chikungunya disease (CCD): clinical insights, immunopathogenesis and therapeutic perspectives

**DOI:** 10.1093/qjmed/hcae028

**Published:** 2024-02-20

**Authors:** W H Ng, K Amaral, E Javelle, S Mahalingam

**Affiliations:** Emerging Viruses, Inflammation and Therapeutics Group, Menzies Health Institute Queensland, Griffith University, Gold Coast, QLD, Australia; Global Virus Network (GVN) Centre of Excellence in Arboviruses, Griffith University, Gold Coast, QLD, Australia; School of Pharmacy and Medical Sciences, Griffith University, Gold Coast, QLD, Australia; Department of Health Sciences, Federal University of Cariri, Barbalha, Ceará, Brazil; Unité Parasitologie et Entomologie, Département Microbiologie et Maladies Infectieuses, Institut de Recherche Biomédicale des Armées, Marseille, France; Aix Marseille Université, IRD, SSA, AP-HM, VITROME, Marseille, France; Unit of Infectious Diseases and Tropical Medicine, IHU Méditerranée Infection, Marseille, France; Service de Pathologie Infectieuse et Tropicale, Hôpital d'Instruction des Armées Laveran, Marseille, France; Emerging Viruses, Inflammation and Therapeutics Group, Menzies Health Institute Queensland, Griffith University, Gold Coast, QLD, Australia; Global Virus Network (GVN) Centre of Excellence in Arboviruses, Griffith University, Gold Coast, QLD, Australia; School of Pharmacy and Medical Sciences, Griffith University, Gold Coast, QLD, Australia

## Abstract

Chikungunya virus, an arthropod-borne pathogen is recognized by the World Health Organization as a top priority Emerging Infectious Disease and is ranked fourth in public health needs according to the Coalition for Epidemic Preparedness Innovations. Despite its substantial impact, as evidenced by an annual estimate of 120 274 disability-adjusted life years, our understanding of the chronic aspects of chikungunya disease remains limited. This review focuses on chronic chikungunya disease, emphasizing its clinical manifestations, immunopathogenesis, therapeutic options and disease burden.

## Introduction

Chikungunya virus (CHIKV), an arthritogenic alphavirus transmitted by mosquitoes, poses a global health threat with its widespread distribution. The virus maintains its existence through a sylvatic cycle involving non-human primates or mammalian host reservoirs, typically with *Aedes* mosquitoes acting as vectors for transmission.^1^ First isolated in 1952 in Tanzania^2^ from a febrile patient, CHIKV has manifested sporadic outbreaks, gaining increased frequency since the early 2000s. This surge started in the previously non-endemic Indian Ocean region and was attributed to the E1-A226V mutation in a strain from the East/Central/South African (ECSA) lineage, enhancing its fitness in *Aedes albopictus* mosquitoes.^1^^,^^2^ This emerging Indian Ocean lineage (IOL) has led to major outbreaks, such as the one in La Réunion in 2006 with an estimated 260 000 cases, and in India during 2005–06 with a reported 1.3 million cases.^1^^,^^2^ However, this figure is believed to be much higher due to the poor reporting of cases in India, particularly in rural regions. In the late 2013, the Asian lineage, which was still circulating in South-East Asia, was introduced to Saint Martin Island and to date, cumulative cases have exceeded 3.6 million across 50 countries in the Americas, with Brazil representing approximately 45% of all reported cases in the continent in the last 10 years.^3^^,^^4^

Infected individuals typically experience acute manifestations, including fever, severe joint and muscle pain, headache, and maculopapular rashes.^1^ The disease is usually self-limiting; however, there is an estimated 40% of CHIKV-infected patients developing disabling arthralgia lasting for more than 3 months after the acute phase of chikungunya disease (CHIKD) which is referred as chronic chikungunya disease (CCD).^5^ CCD is a debilitating major public health concern that not only limits day-to-day activities and reduces the quality of life of affected individuals but also imposes a heavy economic burden.^6^ This review focuses on CCD, an area in medicine where significant gaps in our understanding of the disease still exist, with an emphasis on its clinical manifestations, immunopathogenesis, therapeutic options and global burden.

## Clinical evolution from acute to chronic chikungunya disease

CHIKD is marked by distinct stages in its clinical progression. The acute phase of CHIKD can be separated into two stages: the viraemic phase, lasting for 5–10 days, and the subsequent post-viraemic phase, extending over a period of 6–21 days.^7^ In prevailing clinical presentations, the viraemic phase is characterized by an abrupt onset of high-grade fever (>39°C), accompanying with incapacitating polyarthralgia and at times polyarthritis, myalgia, headache and cutaneous rash.^8^ The post-viraemic phase is typified by apyrexia, the sustained presence of polyarthralgia and arthritis, and, to a lesser extent, myalgia, pruritus, soft tissue oedema, fatigue, lymphadenopathies and anorexia.^9^ Joint pathology typically follows a symmetrical and additive pattern, predominantly affecting both major and minor articulations of the upper limbs—primarily the wrists, followed by the phalanges, shoulders and elbows.^9^ Conversely, in the lower limbs, the ankles are most frequently affected, followed by the knees, feet and hips. Atypical manifestations may include involvement of vertebral, temporomandibular or sternoclavicular articulations.^9^ Significantly, manifestations of stiffness and swelling, indicative of synovitis that may be confirmed by joint ultrasound, are observed prominently in the ankles, phalanges, wrists and toes, with the larger joints exhibiting such features only in rare cases. Moreover, tenosynovitis and bursitis may add to the rheumatic pattern of acute CHIKD.^9^

**Figure 1.**
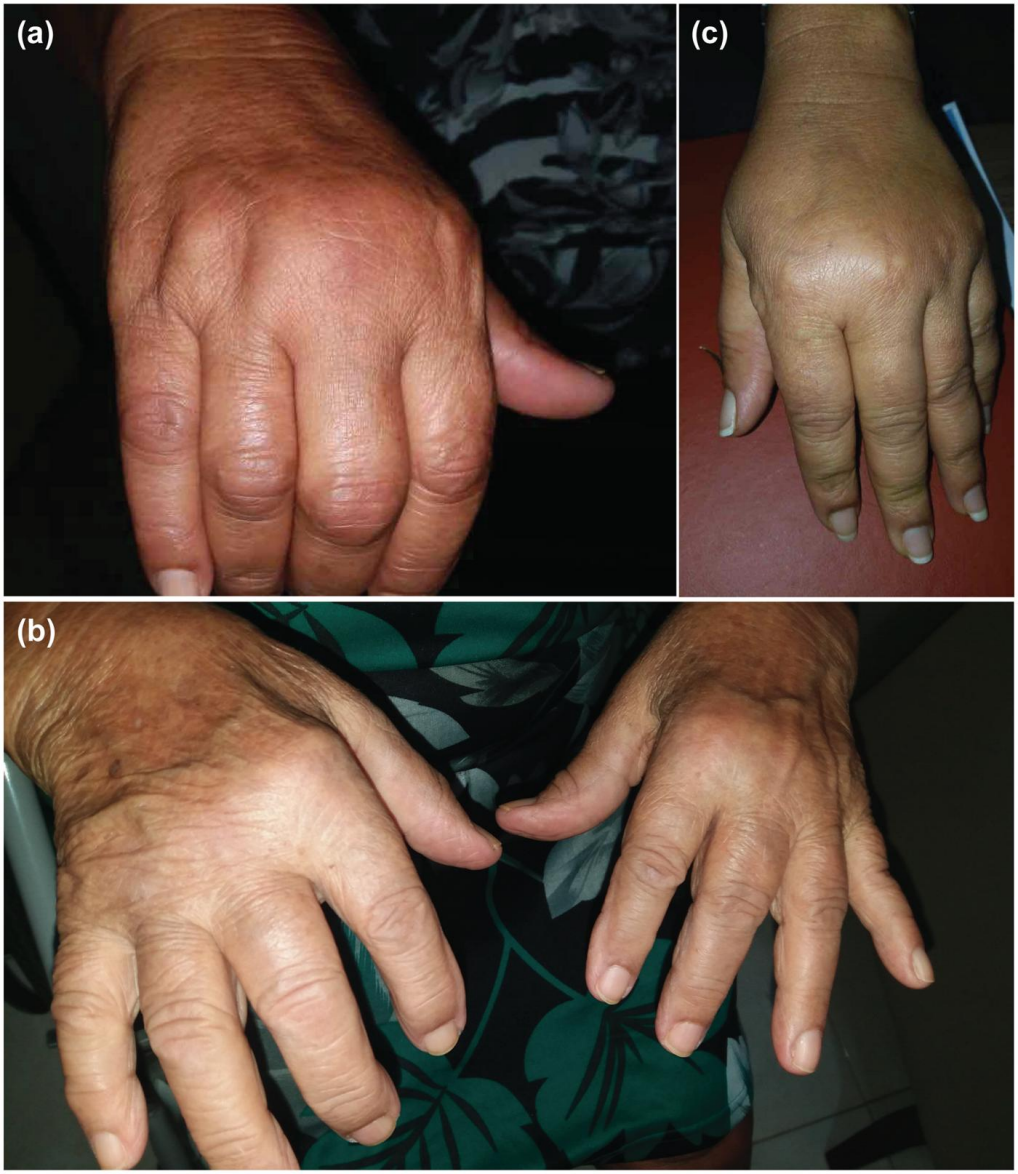
(**a**) Acute chikungunya arthritis in the metacarpophalangeal and proximal interphalangeal joints one week after CHIKV infection, associated with high fever, myalgia, skin rash and headache. (**b**) Chronic chikungunya arthritis affecting proximal interphalangeal, metacarpophalangeal and bilateral wrist joints in a 70-year-old woman 2 years after CHIKV infection. (**c**) Patients developed joint swelling in the hands. Brazilian patients during chikungunya fever outbreaks and serologically confirmed. The images in this manuscript were provided by Dr Kennedy Amaral, the attending physician, and have been authorized for use by both the patients and Dr Amaral.

Typically, CHIKD resolves on its own; however, an estimated 40% of CHIKV-infected patients proceed to a secondary phase characterized by persistent arthritic manifestations.^5^ CCD is defined when symptoms persist beyond 3 months, with rare severe cases persisting for 6 years or for life when CHIKD is the start of chronic inflammatory rheumatism.^10^ In the chronic phase, CCD commonly presents with incapacitating musculoskeletal pains, sometimes with polyarthritis, affecting joints (Figure 1) previously involved during the acute phase, and may extend to additional complications such as stiffness and neuropathic pain. Alopecia, memory and concentration disorders, anxiety and depression are also recognized clinical symptoms associated with CCD.^5^^,^^11^ In some cases, the polyarthritis may meet the 2010 American College of Rheumatology (ACR) and European League Against Rheumatism (EULAR) diagnostic criteria for rheumatoid arthritis (RA)^12–14^ and patients may even develop bone erosions.^5^^,^^11^ Notable reports include Essackjee *et al*.,^12^ in Mauritius with 78.6% of 136 CCD patients reporting persisting musculoskeletal symptoms after 27.5 months post-infection and 5% of them meeting the ACR criteria for RA; Manimunda *et al.*,^13^ in India with 36% of 94 CCD patients meeting the ACR criteria for RA; and Rodriguez-Morales *et al*.,^14^ in Columbia with 89.7% of 39 CCD patients meeting the ACR/EULAR criteria for RA.

The risk factors to progress from acute CHIKD to CCD remains under investigations. Several studies have highlighted that increasing age is associated with increasing risk of CHIKD chronicity.^15^^,^^16^ The role of age as a risk factor for chronicity has also been demonstrated in animal models. In mice, it was shown that aged mice (18–20 months old) had prolonged CHIKV viraemia and evidence of persistent infection in which there was higher viral RNA in joint-associated tissues compared to 12 week-old mice.^16^ In non-human primates’ (NHPs) models of CHIKV infection, it has been shown that viral RNA was detectable in the spleens of aged rhesus macaques, those over 17 years old, but not in adult macaques aged between 6 and13 years.^15^ In clinical studies, risk factors for CCD include being over 40 years of age, experiencing severe arthralgia and arthritis during the acute stage, being female, having co-morbidities such as cardiovascular diseases and rheumatologic disease, and having higher viral loads during the acute phase of infection.^17^^,^^18^ High levels of the cytokine IL-6 and intracellular protein ferritin, during the acute phase are also suggested as markers of risk for CHIKD chronicity.^19^

## Immunopathogenesis of chronic chikungunya disease

The mechanisms underlying CCD are still predominantly unknown and continue to be the focus of ongoing investigations. During the chronic phase, CHIKV viral particles and RNA are typically cleared from the bloodstream; however, studies have indicated the persistence of CHIKV RNA within macrophages and fibroblasts during this stage.^20^^,^^21^ Additionally, viral dsRNA, an intermediate stage in replication, has been detected in the monocytes and macrophages of joints, triggering an arthritogenic response by activating NF-kB. This occurrence has been observed both in *in vitro* studies^22^ and in the ankles and wrists of RAG-1 deficient mice, which lack mature B and T lymphocytes.^23^ The latter study has also suggested that CHIKV viral persistence is genotype independent, and CHIKV viral RNA was still detectable in the ankles of mice even at 4 weeks post-infection when infected with strains SL15649 (ECSA lineage), 37997 (West African lineage), or PO731460 (Asian lineage). However, it should be noted that the amount of viral RNA detected from the CHIKV strains 37997 and PO731460 was significantly higher compared to that from SL15649 strain in infected mice.^23^ Beyond RNA intermediates, the persistence of CHIKV proteins in host cells has been reported over an extended period. CHIKV-nsP3, for instance, was found in granular formations along the cell membrane during persistent infection.^24^ In another study, CHIKV-capsid proteins were detectable in CHIKV-infected mice up to 60 days post-infection, indicating the active translation of viral proteins during the chronic phase of CHIKD.^25^ Despite the identification of viral material such as RNA and/or protein, there have been difficulties in isolating infectious (replication-competent) virus from the joint tissues of patients with subacute or chronic disease.^18^^,^^26^ Potential explanations for this challenge include the presence of low levels of infectious virus or the replication of defective viral RNA as replicons, without the ability to produce infectious virus.^26^

Studies utilizing RNA-seq transcriptional profiling data in mice have suggested that chronic inflammation in CHIKV is essentially an extension of the acute inflammatory response, persisting until viral materials are cleared.^27^ Notably, RNA-seq analysis of CHIKV infection in mice has identified the upregulation of Granzyme A, an activator of pro-inflammatory cytokines.^27^ This observation aligns with data from NHPs and patients with CHIKD. In fact, studies carried out in mouse models have demonstrated that Granzyme A plays a crucial role in RA, highlighting the similarities between CHIKV arthritis and RA.^28^ Furthermore, a meta-analysis conducted revealed abnormal expressions of various pro-inflammatory cytokines and chemokines in CCD patients.^19^ Of note, IL-6, TNF and CCL2 have been identified as the main biomarkers associated with CCD.^19^ This correlation is not surprising, as IL-6, TNF and CCL2 are recognized modulators of the bone remodelling system,^29^ where increased levels of these biomarkers are known to drive osteoclastogenesis, leading to bone loss. This in turn, may contribute to chronic debilitating polyarthralgia—a hallmark of CCD.^5^^,^^11^

In addition to soluble host factors that modulate antiviral responses, CD4+ T cells have been shown as pivotal drivers of arthritic pathology during CHIKV infection in murine models and in longitudinal observations of patients experiencing chronic chikungunya arthritis.^8^^,^^30^^,^^31^ Of notable significance, regulatory T cells (Treg cells), a subset of CD4+ T cells, exhibit a potential role in tempering excessive immune responses induced by CHIKV. In individuals with acute CHIKV infection, a notable decrease in the frequency of peripheral CD4 Treg cells is observed in comparison to naïve controls.^32^ Interestingly, this decrease is reversible, returning to normal levels in patients who did not progress to CCD. On the contrary, those individuals who developed chronic symptoms experienced a sustained reduction in Treg numbers.^32^ However, a report by Wauquier *et al*.^33^ found that the frequency and activation status of peripheral CD4 Treg cells were comparable between patients with acute CHIKV infection and healthy controls. This discrepancy underscores the need for more in-depth investigations to comprehensively understand the role of CD4 Treg cells during both acute and CCD.

The role of natural killer (NK) cells has also been evaluated on CCD. One investigation reported an increased presence of NK cells in peripheral blood mononuclear cells (PBMCs) of patients with CCD compared to healthy controls.^34^ Furthermore, in the same study, it was demonstrated that upon *in vitro* stimulation, the frequency of NK cells expressing perforin and CD107a was diminished in PBMCs from patients with CCD when compared to healthy controls.^34^ This suggests that impaired NK cell function, characterized by reduced cytolytic activity could be associated with CCD. Conversely, there was an elevation in the frequency of NK cells expressing TNF and IFN-γ, indicating an enhanced production of pro-inflammatory cytokines by NK cells in CCD.^34^ These findings suggest the involvement of NK cells in the context of CCD. However, the precise role of NK cells in CCD remains unclear and necessitates further in-depth investigation.

## Management and therapeutic strategies for chronic chikungunya disease

To date, approximately 10 vaccines targeting CHIKV are under development,^35^ one of which, the live attenuated VLA1553 (Ixchiq), was approved by the Food and Drug Administration (FDA) in late 2023. However, uncertainties persist regarding its tolerance and clinical efficacy. Thus, large-scale or targeted immunization programmes are not ready to be fully implemented, while antiviral options remain unavailable.^3^

In the acute phase, paracetamol is the best option for managing the fever and pain caused by the virus. In severe cases, opioids like codeine and tramadol, or even analgesia, may be considered to alleviate pain.^7^ Non-steroidal anti-inflammatory drugs (NSAIDs) are not recommended during the acute phase to avoid disturbing the immune system’s viral clearance processes and to reduce the risk of severe forms. However, they may be considered as soon as the end of the viraemic stage if the patient’s symptoms become intractable and other arboviral infections—mainly dengue in endemic areas are ruled out.^36^

**Figure 2.**
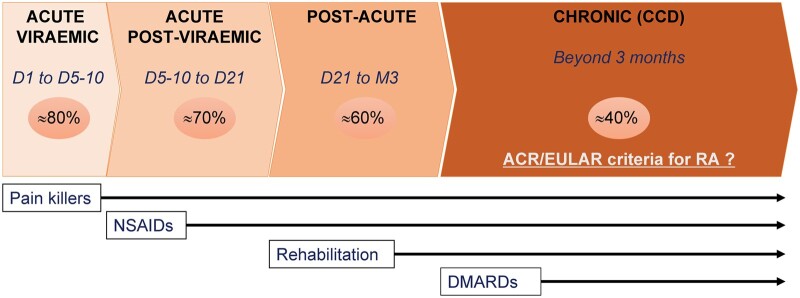
Stages of chikungunya disease are represented with globally estimated prevalence (% in circles), CDD for chronic chikungunya disease, D for days, and M for months. The time for the introduction of therapeutic strategies is represented under the graph with NSAIDs for non-steroidal anti-inflammatory drugs, DMARDs for disease-modifying anti-rheumatic drugs. American College of Rheumatology (ACR) and European League Against Rheumatism (EULAR) are diagnostic criteria for rheumatoid arthritis (RA).

During the chronic phase of CHIKD, patients are advised to consult a rheumatologist or pain specialist for personalized treatment options. Typically, treatment for CCD patients is tailored to their specific clinical phenotype. The initial recommendation often involves the early use of full-dose of NSAIDs in combination with analgesics until the complete resolution of chronic symptoms. Drugs must be associated with physical for analgesia, to regain and maintain flexing amplitudes and muscle tone, and for lymphatic drainage.^7^ Stiffness must be managed by rehabilitation early in the course of the CCD regarding its major functional impact (Figure 2).^37^

**Figure 3.**
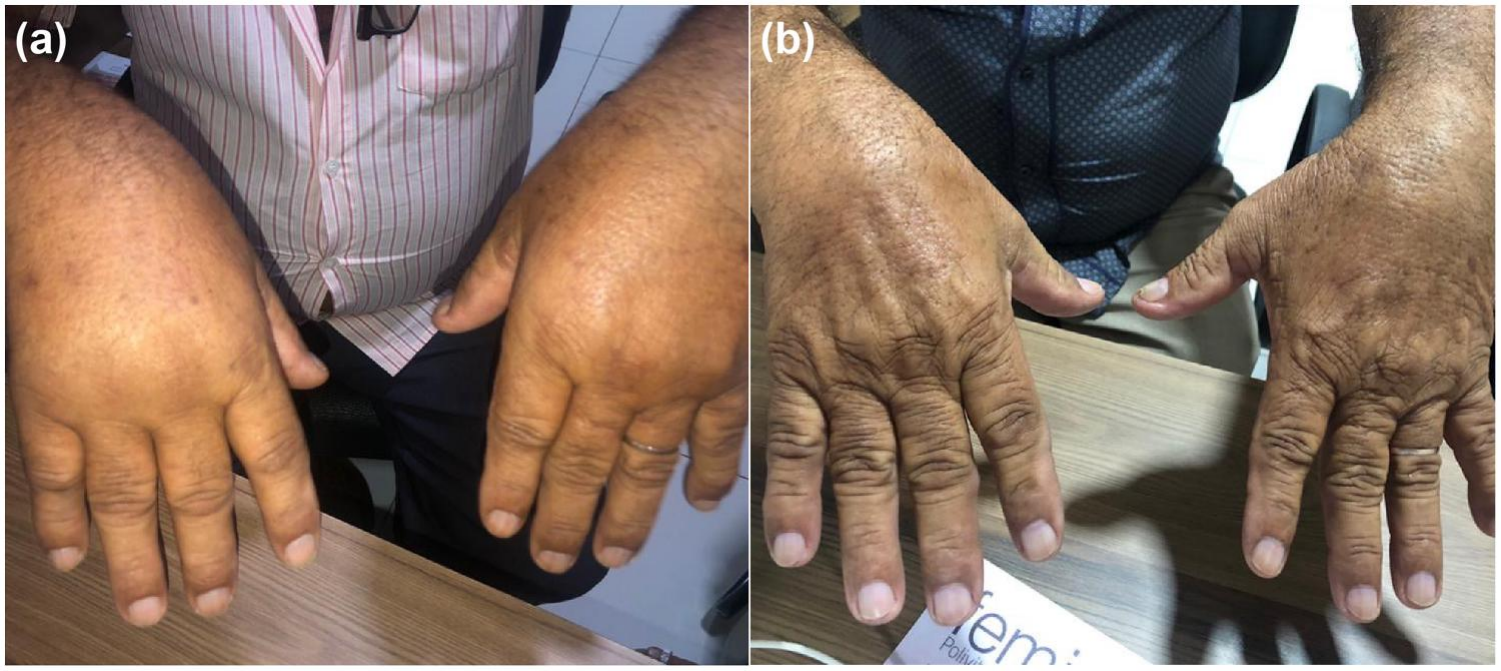
(**a**) A 65-year-old man with symmetrical arthritis in hands 6 months after serologically confirmed CHIKV infection before treatment with Methotrexate and (**b**) 15 days after starting treatment. The images in this manuscript were provided by Dr Kennedy Amaral, the attending physician, and have been authorized for use by both the patient and Dr Amaral.

If patients do not respond to NSAIDs, other therapeutic options will be explored.^7^^,^^36^ Given the similarities in clinical symptoms between CCD and RA patients, disease-modifying anti-rheumatic drugs (DMARDs), traditionally used to treat rheumatological symptoms are commonly used to treat CCD with hydroxychloroquine (HCQ) being the preferred first-line option.^38^ Methotrexate (MTX) is only recommended (sometimes used in combination with HCQ) if there is a case of severe persistent inflammatory polyarthralgia and arthritis (disease affecting >5 joints) and positive outcomes have been associated with this approach (Figure 3).^38–40^ Clinical trials exploring the use of DMARDs in treating CCD has been published; however, evidence regarding their efficacy remains inconclusive.^7^^,^^36^ The varied success rates reported and challenges in drawing definitive conclusions arise from the diverse agents employed and the heterogeneity of patient characteristics.^38^^,^^39^^,^^41^ One study has reported a positive outcome in 75% CCD patients treated with MTX but 27% of the patients experienced adverse effects with seven patients experiencing bone erosions, of which six eventually recovered from this complication.^40^ Amaral *et al*.^38^^,^^39^^,^^42^ reported numerical improvement trends in CCD patients treated with MTX with or without combination treatment with dexamethasone in three separate studies.

Based on preclinical findings, future clinical trials will be necessary to investigate the targeting of specific cytokines or host factors potentially linked to CCD. For example, studies have demonstrated that patients with CCD exhibit significantly increased levels of IL-17A compared to healthy individuals, and CHIKV-infected mice lacking IL-17A showed reduced foot swelling at the post-acute stage compared to their wild-type counterparts, indicating a role for IL-17A in the later stages of the disease.^43^ Therefore, drugs targeting IL-17A might be worth investigating as a potential treatment for CCD.^43^ We recently identified a crucial interaction between CHIKV non-structural protein 3 (nsP3) and four-and-a-half-LIM domain protein 1 (FHL1), a host protein that is important for muscle growth and essential for CHIKV replication.^44^ FHL1 expression is also highly upregulated in patients with both acute and chronic CHIKV infections. Since the FHL1-nsP3 interaction is crucial for the optimal growth of CHIKV, both these host and viral factors may be prerequisites for persistent infection that could be linked to chronic disease. Future work focusing on developing therapeutics that target the interaction between FHL1 and nsP3 could provide potential treatment options for CCD. The use of glycosaminoglycan holds promise for treating CCD, as pentosan polysulfate (PPS), an FDA-approved drug for interstitial cystitis, has shown promising results in preclinical studies and human trials.^45^^,^^46^ In phase 2a clinical trials, PPS treatment showed positive outcomes for patients with severe Ross River virus-induced arthralgia. Those treated with PPS demonstrated improved grip strength, reduced overall joint symptoms, and favourable levels of six biomarkers related to bone integrity, compared to patients who received a placebo.^46^

## Cost and functional burden of chronic chikungunya disease

CCD significantly impacts global economic costs, with the calculated disability-adjusted life years (DALYs) for CHIKD from 2011–20 equates to 120 274 years lost annually.^6^ However, this figure is likely an underestimation due to incomplete surveillance and underreporting before 2014.^6^ Unsurprisingly, most DALY burden is a result of CCD accounting to 76% of the calculated score. The collective global cost of chikungunya between 2011–20 is estimated at 46.7 billion USD.^6^ Even in travellers returning with CHIKD, the cost of illness is higher than with other travel-acquired vector-borne diseases, with a median loss of 2400 USD (IQR 1200–3600).^47^ Studies evaluating patients’ quality of life (QoL) using the 12-Item Short-Form Health Survey (SF-12) questionnaire, or the disability in CHIKD patients using the Health Assessment Questionnaire Disability Index (HAQ-DI), are summarized in a study by Amaral *et al*.^48^ This study highlights the negative outcomes directly attributed to chronic CHIKV infection, reporting scores that correlate with high pain levels, reduced daily activities, functional disabilities and poor mental health.^48^

## Conclusion

CCD stands as a critical global health concern, acknowledged as a priority by healthcare experts. A notable disparity in public awareness and funding support for research in this area has led to a lack of attention and resources, hampering advancements in understanding the mechanisms, immunopathogenesis, risk factors and clinical evolution of CCD. A comprehensive understanding of CCD is essential for the development of targeted therapeutics aimed at mitigating its global societal and economic impact. Increasing awareness among the public, government and non-governmental organizations is crucial to gaining the necessary support for more research focused on combating the challenges posed by CCD.
